# Acute kidney injury with partial Fanconi syndrome in a patient with leptospirosis: a case report

**DOI:** 10.1186/s13256-021-02978-0

**Published:** 2021-07-23

**Authors:** Marc Weiner, Matteo Coen, Jacques Serratrice, Thomas A. Mavrakanas, Antonio Leidi

**Affiliations:** 1 Department of General Internal Medicine, Geneva University Hospital, Rue Gabrielle-Perret-Gentil 4, 1205 Geneva, Switzerland; 2 Division of Nephrology, Department of Medicine, McGill University Health Centre, 1001 Decarie Boulevard, Montreal, QC Canada; 3 Unit of Development and Research in Medical Education (UDREM), Faculty of Medicine, University of Geneva, 1211 Geneva, Switzerland

**Keywords:** Leptospirosis, Kidney injury, Fanconi syndrome, Proximal tubular dysfunction, Glucose, Case report

## Abstract

**Background:**

Leptospirosis is an underdiagnosed bacterial infection with nonspecific symptoms, hence, a diagnostic challenge. Identifying a case of leptospirosis in Switzerland is uncommon. Although kidney complications are frequent in severe forms, including tubular dysfunction, observing this complication is rare in our country. We report the case of a patient with leptospirosis and kidney dysfunction, which was notable for proximal tubulopathy. This case report describes the diagnosis and management of this patient’s tubular dysfunction.

**Case presentation:**

A 34-year-old Caucasian male known for alcohol and drug abuse presented to our emergency department suffering from severe pain in the lower limbs, jaundice, and fever with flu-like symptoms. Physical examination was not contributory. Blood tests showed cytopenia, elevated inflammatory markers, acute kidney injury, and altered liver function tests with predominant cholestasis. Urinalysis showed proteinuria and significant glycosuria without concomitant hyperglycemia. Leptospirosis was suspected and confirmed by both positive serum polymerase chain reaction and elevated immunoglobulin M for *Leptospira interrogans*. The patient was treated with intravenous amoxicillin–clavulanate and doxycycline for 7 days. After antibiotic treatment, symptoms disappeared, and kidney dysfunction completely resolved.

**Conclusion:**

Our case focuses on the description of leptospirosis-related acute kidney injury with proximal tubular dysfunction, which is a rare finding in Switzerland.

## Background

Leptospirosis is a zoonotic bacterial infection caused by the spirochete *Leptospira interrogans*. It is transmitted to humans through water or soil contaminated by the urine of infected mammals, most commonly rodents. Contamination occurs by inhalation or ingestion, or through a breach in the skin. Symptoms vary from mild illness with self-limited fever to life-threatening multiorgan dysfunction [1]. Antibiotic therapy with beta-lactams, doxycycline, or macrolides is the mainstay of treatment [2]. In Switzerland, infections mostly occur during summer and fall, probably because the climate favors spirochete growth, and aquatic activities in rivers or lakes increase during the warm season [3]. In Switzerland, 2–13 cases were reported yearly between 1988 and 1996, corresponding to the last year of mandatory reporting for human infections [4]. According to the Swiss Federal Food Safety and Veterinary Office, mortality rate is about 20% for severe forms [5], referred to as Weil’s disease, which occurs in 5–10% of patients. Clinical manifestations include jaundice, thrombocytopenia, respiratory symptoms, myocarditis, conjunctival suffusion, and kidney impairment [6].

Kidney complications include acute kidney injury (44–67% of patients [7]), commonly due to tubulointerstitial nephritis, and tubular dysfunction. The latter has been associated with hypophosphatemia, hypokalemia, hypouricemia, and metabolic acidosis, mimicking Fanconi syndrome [8, 9], which represents impaired reabsorption in the proximal tubule of the nephron, resulting in loss of bicarbonate, glucose, phosphate, uric acid, and amino acids [10]. We report herein a case of acute kidney injury with partial Fanconi syndrome in a leptospirosis-infected patient.

## Case presentation

A 34-year-old Caucasian male presented to the emergency department complaining of a 4-day history of unbearable leg pain with diffuse arthralgia, fluctuating low-grade fever with profuse sweating, vomiting and diarrhea without abdominal pain, odynophagia, dry cough, headaches, and fatigue. He denied any contact with animals or travel abroad, had not consumed unpasteurized food, and had his last unprotected sexual intercourse 4 months earlier.

He was taking no medications and was known for alcohol abuse (mainly beer and spirit, approximately 185 alcohol units/week), drug abuse (cocaine, ecstasy, cannabis, methylphenidate, clonazepam, lorazepam), and smoking tobacco. On physical examination, he was afebrile and hypotensive (blood pressure 94/50 mmHg) with a normal heart rate (77 beats/minute). Oral examination revealed dry mucosa and erythematous tonsils without exudate, cardiopulmonary examination was normal, abdominal palpation was unremarkable, and no cutaneous rash was noted. Testing of the lower limbs revealed preserved strength and sensitivity as well as symmetric deep tendon reflexes.

Laboratory findings showed normocytic, normochromic, hypoproliferative anemia (hemoglobin 99 g/l, normal range 140–180 g/l) with thrombocytopenia (24 G/l, normal range 150–350 G/l), left shift without leukocytosis, elevated C-reactive protein (213 mg/l, normal range < 10 mg/l), elevated transaminases (three times the upper limit of normal) with cholestasis and elevated conjugated bilirubin (29 µmol/l on admission, 190 µmol/l on hospital day 8, normal range 0.5–9.5 µmol/l). There was a stage 3 acute kidney injury according to the Kidney Disease Improving Global Outcomes (KDIGO) criteria with a serum creatinine of 112 µmol/l on admission and 247 µmol/l on hospital day 3 (patient’s baseline value 70 µmol/l), a serum urea of 11.4 mmol/l on admission (12 mmol/l on hospital day 3), and preserved urine output. Urinalysis revealed proteinuria (spot urine: protein 1.4 g/l, creatinine 11.5 mmol/l, urine protein–creatinine ratio 1.076 g/g), albuminuria (2+ on semiquantitative analysis), presence of 97 M/l leukocytes, 25 M/l erythrocytes, and renal tubular cells (1+ on semiquantitative analysis). Sodium excretion was high (fractional excretion of sodium on spot urine 3.56%). Due to the context of sepsis, hypovolemic status of the patient, and urinalysis findings, acute tubular necrosis was the suspected mechanism of acute kidney injury.

Analysis of serum electrolytes showed mild hyperphosphatemia (1.53 mmol/l, normal range 0.80–1.45 mmol/l), hyponatremia (131 mmol/l, normal range 136–144 mmol/l), mild hypouricemia (246 µmol/l, normal range 286–518 µmol/l), low serum chloride (94 mmol/l, normal range 98–106 mmol/l), hypokalemia (2.8 mmol/l, normal range 3.6–4.6 mmol/l), and an anion gap of 10 mmol/l. The transtubular potassium gradient was elevated (16.4). Albumin level was 29 g/l (normal range 35–48 g/l), and creatinine kinase level was 312 U/l on admission, rising to 404 U/l the next day and normalizing on hospital day 4. Blood gas analysis showed a pH of 7.49 (normal range 7.35–7.45) with elevated bicarbonates (29.3 mmol/l, normal range 22–26 mmol/l) and normal pCO_2_ (5.25 kPa, normal range 4.7–6.4 kPa). Urine pH was 6 (normal range 5–6.5). Presence of glycosuria (7 mmol/l, normal range 0.1–0.9 mmol/l), with concomitant euglycemia (6.6 mmol/l, normal range 4.1–11 mmol/l) and a hemoglobin A1c of 5.1% was highly suspicious of proximal tubular dysfunction.

Upon admission, Streptococcus A rapid test was negative, chest radiograph was normal, and an abdominal ultrasound showed hepatomegaly, a heterogeneous parenchyma with hyperechogenicity around periportal spaces and lymph nodes, liver parenchymal arterialization, and a layer of perihepatic fluid, all consistent with hepatitis. The bile ducts were not dilated. Kidneys were of normal size and morphology, without any urinary tract dilatation. On the second day after admission, the patient developed drowsiness and confusion with high fever (exceeding 40 °C). Normal cerebral magnetic resonance imaging and lumbar puncture excluded central nervous system infection. Clinical features were suggestive of bacterial sepsis [sequential organ failure assessment [SOFA] score of 10], justifying empiric broad spectrum antibiotic therapy with intravenous ceftriaxone and oral clarithromycin.

Detailed history revealed that the patient swam in a river in the Geneva lake area 1 week before hospital admission, raising the diagnostic suspicion of leptospirosis in this clinical setting. Serum polymerase chain reaction (PCR) for *Leptospira interrogans* was positive, and specific serologies revealed elevated IgM levels (> 100 U/ml, normal range < 15 U/ml). Although IgG levels were below the positive threshold, the values increased from < 2 U/ml initially to 6 U/ml 1 week later (normal range < 10 U/ml). Unfortunately, no ulterior dosage was performed. Antibiotic therapy was changed to intravenous amoxicillin–clavulanate and doxycycline for 7 days with clinical improvement. Serology for hantavirus was performed, revealing an indeterminate result due to nonspecific reactions. Considering the positive results for *Leptospira interrogans*, additional tests for hantavirus were not performed.

Along with clinical improvement, kidney function recovered with creatinine normalizing to 87 μmol/l on the sixth hospital day, and all electrolyte abnormalities resolved. Liver tests also normalized as confirmed by a routine blood sample 1 year after discharge. In addition, glycosuria significantly decreased from 7 to 0.5 mmol/l on the 12th day of hospital stay.

**Figure:**
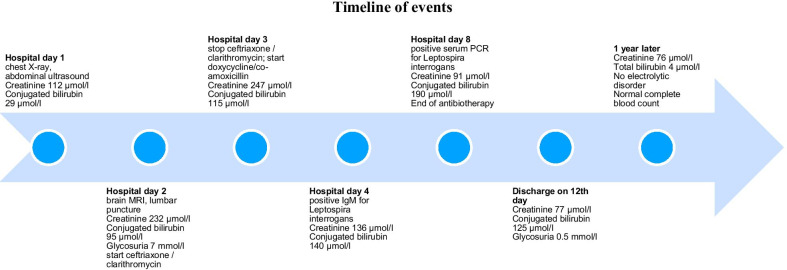

## Discussion and conclusion

We report the case of a patient with leptospirosis who developed acute kidney injury and partial Fanconi syndrome diagnosed on the basis of euglycemic glycosuria. The patient also had proteinuria, although its tubular origin cannot be definitely proven in the absence of a urine albumin-to-creatinine ratio. There was no evidence of proximal renal tubular acidosis. The patient also had mild hyperphosphatemia, but this could be attributed to severe acute kidney injury. Concerning hypokalemia with an increased transtubular gradient, it is most likely explained by an elevated aldosterone level in the context of sepsis and hypovolemia.

The alkalosis observed is unusual in the setting of proximal tubulopathy and acute kidney injury. Considering the context of hypovolemia and sepsis, without hypocapnia, contraction alkalosis is an explanation. Hypokalemia and metabolic alkalosis may also have been caused by the vomiting.

In humans, leptospirosis-induced kidney tubular dysfunction is mostly observed in the proximal tubule [8]. Some reports of proximal tubular dysfunction in leptospirosis-infected patients described hypokalemia, hypophosphatemia, hypouricemia, glycosuria, and metabolic acidosis [11–14].

Animal models have confirmed that *Leptospira interrogans* can infect kidney proximal tubular epithelial cells through hematogenous spread. Spirochetes enter cells through the basal membrane and then translocate to the apical membrane where they form a biofilm-like structure enabling them to resist the urinary stream [15]. Leptospirosis also inhibits expression of electrolytes transporters in nephrons. The outer membrane protein (OMP) of spirochetes inhibits mRNA synthesis of the sodium, potassium, and chloride cotransporter (NKCC2) [16]. Antibiotic treatment results in an increased activity of the transporters [17]. Other molecular mechanisms of tubular injury in leptospirosis involve the sodium–hydrogen exchanger type 3 (NHE3) [17], Toll-like receptor-dependent pathway [18], and nuclear factor kappa-B (NFkB) [19]. A detailed description of these mechanisms is beyond the scope of this manuscript.

**Fig. 1:**
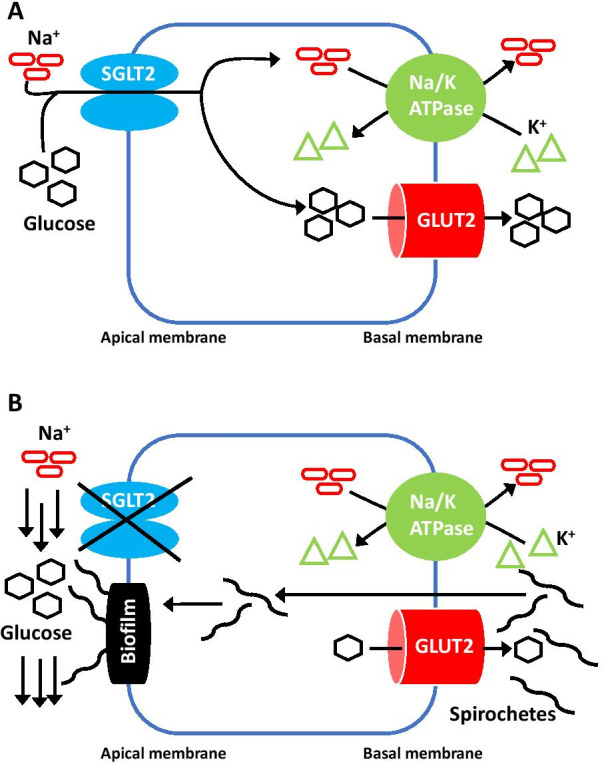
Mechanism of glucose reabsorption in the proximal tubule of the nephron (**A**) and its impairment in leptospirosis (**B**). The mechanism is similar for SGLT1 in the distal segment of the proximal tubule

As glucose is reabsorbed in the proximal tubule by the sodium–glucose cotransporter 2 and, to a lesser extent, sodium–glucose cotransporter 1 (SGLT2, SGLT1) (Fig. 1A), the euglycemic glycosuria developed by our patient suggests a potential inhibition of SGLT2 and/or SGLT1 by spirochetes (Fig. 1B).

Although acute interstitial nephritis is part of the differential diagnosis of tubulopathy, we do not think it is the most likely explanation owing to the clinical presentation, the urine sediment findings (tubular cells), and the absence of white blood cell casts.

Although alcohol and drug abuse are known to induce Fanconi syndrome [20], our patient was reportedly abstinent during the week prior to hospital admission, and a toxicology screening at the beginning of the hospitalization was only positive for benzodiazepines, which the patient was given in hospital previously to the screening. Moreover, glycosuria resolved after antibiotic treatment, thus favoring our hypothesis that the partial Fanconi syndrome was secondary to leptospirosis rather than substance abuse.

To conclude, the clinical manifestations of leptospirosis are numerous and nonspecific, which possibly leads to misdiagnosis, especially in Switzerland, where this infection is seldom reported. Among the known clinical manifestations, we focused on kidney tubular dysfunction—specifically, impaired proximal reabsorption of glucose. Our case illustrates the occurrence of transient euglycemic glycosuria in the setting of leptospirosis, which resolved after appropriate antibiotic therapy.

## Data Availability

Data sharing is not applicable to this article as no datasets were generated or analyzed during the current study.

## Abbreviations

PCR: Polymerase chain reaction
IgM/IgG: Immunoglobulin M/immunoglobulin G
KDIGO: Kidney Disease Improving Global Outcomes
pCO_2_: Partial pressure of carbon dioxide
SOFA: Sequential organ failure assessment
OMP: Outer membrane protein
mRNA: Messenger ribonucleic acid
NKCC2: Sodium, potassium, and chloride cotransporter
NHE3: Sodium–hydrogen exchanger type 3
NFkB: Nuclear factor kappa-B
SGLT1 and 2: Sodium–glucose cotransporter 1 and 2

## References

[1] Lane AB, Dore MM. Leptospirosis: a clinical review of evidence based diagnosis, treatment and prevention. World J Clin Infect Dis. 2016;6:61–6. 10.5495/wjcid.v6.i4.61.

[2] Johnson RC, Faine S. Leptospira. In: Bergey’s manual of systematic bacteriology, vol. 1. 1984:62–7.

[3] Schreiber PW, Aceto L, Korach R, *et al*. Cluster of leptospirosis acquired through river surfing in Switzerland. Open Forum Infect Dis. 2015;2:ofv102. 10.1093/ofid/ofv102. PMC4531225 26269796

[4] Giulieri S, Tissot F. A short journey through spirochetal infections. Rev Med Suisse. 2010;6:721–6. 20432993

[5] Federal Food Safety and Veterinary Office. La leptospirose chez l'homme et chez l'animal. 2017. https://www.blv.admin.ch/blv/fr/home/tiere/tierseuchen/uebersicht-seuchen/alle-tierseuchen/leptospirose.html. Accessed 14 Sept 2020.

[6] Levett PN. Leptospirosis. Clin Microbiol Rev. 2001;14:296–326. 10.1128/CMR.14.2.296-326.2001. PMC88975 11292640

[7] Cetin BD, Harmankaya O, Hasman H, *et al*. Acute renal failure: a common manifestation of leptospirosis. Ren Fail. 2004;26:655–61. 10.1081/jdi-200037154. 15600257

[8] Khositseth S, Sudjaritjan N, Tananchai P, *et al*. Renal magnesium wasting and tubular dysfunction in leptospirosis. Nephrol Dial Transplant. 2008;23:952–8. 10.1093/ndt/gfm698. 17951309

[9] Picardeau M. Virulence of the zoonotic agent of leptospirosis: still terra incognita? Nat Rev Microbiol. 2017;15:297–307. 10.1038/nrmicro.2017.5. 28260786

[10] Izzedine H, Launay-Vacher V, Isnard-Bagnis C, *et al*. Drug-induced Fanconi’s syndrome. Am J Kidney Dis. 2003;41:292–309. 10.1053/ajkd.2003.50037. 12552490

[11] Liamis G, Rizos E, Elisaf MS. Reversible proximal tubular dysfunction in a patient with acute febrile illness and normal renal function: an evidence towards leptospirosis. Clin Nephrol. 2000;53:316. 10809424

[12] Liberopoulos E, Bairaktari E, Elisaf M. Reversible proximal tubular dysfunction in a patient with acute febrile illness, marked hyperbilirubinemia and normal renal function: evidence of leptospirosis. Nephron. 2002;91:532–3. 10.1159/000064304. 12119494

[13] Yang CW, Pan MJ, Wu MS, *et al*. Leptospirosis: an ignored cause of acute renal failure in Taiwan. Am J Kidney Dis. 1997;30:840–5. 10.1016/s0272-6386(97)90091-3. 9398130

[14] Krishnan A, Karnad DR, Medhekar TP. Paralysis due to renal potassium wasting: an unusual presentation of leptospirosis. Nephrol Dial Transplant. 2003;18:2454–5. 10.1093/ndt/gfg350. 14551392

[15] Yamaguchi T, Higa N, Okura N, *et al*. Characterizing interactions of *Leptospira interrogans* with proximal renal tubule epithelial cells. BMC Microbiol. 2018;18:64. 10.1186/s12866-018-1206-8. PMC6030750 29973159

[16] Wu MS, Yang CW, Pan MJ, *et al*. Reduced renal Na+-K+-Cl- co-transporter activity and inhibited NKCC2 mRNA expression by Leptospira shermani: from bed-side to bench. Nephrol Dial Transplant. 2004;19:2472–9. 10.1093/ndt/gfh452. 15388818

[17] Spichler A, Ko AI, Silva EF, *et al*. Reversal of renal tubule transporter downregulation during severe leptospirosis with antimicrobial therapy. Am J Trop Med Hyg. 2007;77:1111–9. 18165532

[18] Yang CW. Leptospirosis renal disease: understanding the initiation by Toll-like receptors. Kidney Int. 2007;72:918–25. 10.1038/sj.ki.5002393. 17687261

[19] Yang CW, Wu MS, Pan MJ, *et al*. Leptospira outer membrane protein activates NF-kappaB and downstream genes expressed in medullary thick ascending limb cells. J Am Soc Nephrol. 2000;11:2017–26. 10.1681/ASN.V11112017 11053477

[20] Werion A, Lengele JP. Fanconi syndrome in alcoholic patients: old concept new idea. Nephron. 2019;141:201–2. 10.1159/000496035. 30602151

