# Correction to: Acute kidney injury with partial Fanconi syndrome in a patient with leptospirosis: a case report

**DOI:** 10.1186/s13256-021-03040-9

**Published:** 2021-08-16

**Authors:** Marc Weiner, Matteo Coen, Jacques Serratrice, Thomas A. Mavrakanas, Antonio Leidi

**Affiliations:** 1 Department of General Internal Medicine, Geneva University Hospital, Rue Gabrielle-Perret-Gentil 4, 1205 Geneva, Switzerland; 2 Division of Nephrology, Department of Medicine, McGill University Health Centre, 1001 Decarie Boulevard, Montreal, QC Canada; 3 Unit of Development and Research in Medical Education (UDREM), Faculty of Medicine, University of Geneva, 1211 Geneva, Switzerland

## Correction to: J Med Case Reports (2021) 15:358 https://doi.org/10.1186/s13256-021-02978-0

Following publication of the original article [1], the paragraph in the top right part and the paragraph in bottom right part of the page 3 have been interchanged due to a typesetting error.

The original article [1] has been corrected.
